# MicroRNAs as Immunotherapy Targets for Treating Gastroenterological Cancers

**DOI:** 10.1155/2018/9740357

**Published:** 2018-06-26

**Authors:** Yixin Yang, Christopher Alderman, Ayoub Sehlaoui, Yuan Xiao, Wei Wang

**Affiliations:** ^1^College of Natural, Applied and Health Sciences, Kean University, 100 Morris Avenue, Union, NJ 07083, USA; ^2^School of Medicine, University of Colorado, 13001 E 17th Pl, Aurora, CO 80045, USA; ^3^Department of Biological Sciences, Emporia State University, 1 Kellogg Circle, Emporia, KS 66801, USA; ^4^Department of Thoracic Surgery III, Cancer Hospital of China Medical University, No. 44 Xiaoheyan Road, Dadong District, Shenyang, Liaoning 110042, China

## Abstract

Gastroenterological cancers are the most common cancers categorized by systems and are estimated to comprise 18.4% of all cancers in the United States in 2017. Gastroenterological cancers are estimated to contribute 26.2% of cancer-related death in 2017. Gastroenterological cancers are characterized by late diagnosis, metastasis, high recurrence, and being refractory to current therapies. Since the current targeted therapies provide limited benefit to the overall response and survival, there is an urgent need for developing novel therapeutic strategy to improve the outcome of gastroenterological cancers. Immunotherapy has been developed and underwent clinical trials, but displayed limited therapeutic benefit. Since aberrant expressions of miRNAs are found in gastroenterological cancers and miRNAs have been shown to regulate antitumor immunity, the combination therapy combining the traditional antibody-based immunotherapy and novel miRNA-based immunotherapy is promising for achieving clinical success. This review summarizes the current knowledge about the miRNAs and long noncoding RNAs that exhibit immunoregulatory roles in gastroenterological cancers and precancerous diseases of digestive system, as well as the miRNA-based clinical trials for gastroenterological cancers. This review also analyzes the ongoing challenge of identifying appropriate therapy candidates for complex and dynamic tumor microenvironment, ensuring efficient and targeted delivery to specific cancer tissues, and developing strategy for avoiding off-target effect.

## 1. Introduction

Gastroenterological cancers are most common cancers categorized by systems and are estimated to comprise 18.4% of all cancers in the United States in 2017 [1]. Gastroenterological cancers constitute a leading cause of cancer-related deaths, contributing 26.2% of estimated cancer death in 2017. Colorectal cancer, liver and intrahepatic bile carcinoma, and pancreatic cancer continue to be ranked as three of the top 10 cancers with the largest number of new cases and deaths [1]. Most gastroenterological cancers, especially hepatocellular carcinoma and pancreatic cancer, are characterized by latent disease course, metastasis, high recurrence, and being refractory to current therapies. Therefore, gastroenterological cancers are often associated with poor prognosis.

With better understanding of molecular mechanisms of carcinogenesis, cell self-renewal and uncontrolled growth, metastasis, and other landmarks of cancer, progress has been made in developing and obtaining FDA approval of biological therapies targeting oncogenic signaling driver molecules including vascular-endothelial growth factor (VEGF) and its receptor VEGF-R [2], epidermal growth factor receptor (EGFR) [3], and human epidermal growth factor receptor-2 (HER2/Neu) [4]. The monoclonal antibodies antagonizing these cell growth driver molecules have achieved improved response rate (RR), progression-free survival (PFS), and overall survival (OS), demonstrating varying levels of success in colorectal and gastroesophageal cancers. However, the response is not durable and resistance is almost inevitably developed due to both innate and acquired mechanisms [5]. Gemcitabine, a standard treatment choice for advanced pancreatic cancer, produces only modest effect on survival (5.65 months versus 4.41 months) [6]. The very limited clinical efficacy is attributed to poor cell uptake of the drug, dense fibrous tumor stroma, and the development of gemcitabine resistance [7]. Sorafenib, a multikinase inhibitor, is the only FDA-approved drug for metastasized HCC, improving overall survival by only 2.8 months [8]. Since the current therapies provide limited benefit to the overall response and survival and are susceptible to resistance development, there is pressing need for developing novel therapeutic strategies to improve the outcome of gastroenterological cancers.

The aberrant expressions of genetically or epigenetically altered proteins in cancers produce cancer specific antigens. Since the cancer specific antigens were discovered in melanoma in 1990s, cancer immunotherapy has become a promising treatment strategy that deliberately uses the activated innate immunity and cancer specific adaptive immunity to reject tumors and prevent metastasis and reoccurrence [9]. Cancer immunotherapy employing cancer peptide vaccine [10], adaptive T cell therapies [11], and antibodies modulating regulatory T cells and achieving immunity checkpoint blockage [12] has been extensively studied in both basic research and clinical trials. Immunotherapy aims to induce strong, specific, and persistent anticancer immune response in tumor microenvironment.

It is well understood that tumors develop sophisticated mechanisms to disarm the immune system and evade the immune surveillance. Many cancers can produce or induce the immune cells in tumor stroma to produce an array of immunosuppressive cytokines including transforming growth factor (TGF‐*β*) and IL-10, which inhibit the recruitment and activation of antitumor T lymphocytes [12]. In addition, IL-6 suppresses antigen presentation ability of dendritic cells through activation of signal transducer and transcription activator 3 (STAT3) and attenuates CD4^+^ T cell‐mediated immune responses [13, 14]. Furthermore, immunosuppressive cells including Foxp3^+^ CD4^+^ regulatory T cells (Tregs) and myeloid‐derived suppressor cells (MDSC) in tumor microenvironments play significant roles in suppressing anticancer immunity [15, 16]. Therefore, immunotherapy using monoclonal antibody antagonizing the immunosuppressive cytokines or inactivating immunosuppressive cells can enhance anticancer immunity and inhibit tumor growth [17].

Immunotherapy that has achieved acclaimed clinical success primarily targets on two immune checkpoint molecules: PD-1/PD-L1 and CTLA4. Programmed cell death 1 (PD-1,* pdcd1*) is a type I transmembrane glycoprotein belonging to the CD28/CTLA-4 family. PD-1 is expressed on T cells in thymus and on peripheral B and T cells [18]. PD-L1 and PD-L2 are type I transmembrane glycoprotein and serve as the ligands of PD-1. PD-L1 and -L2, especially PD-L1, are extensively expressed in both lymphoid and nonlymphoid tissues [19, 20], suggesting that PD-1-PD-L1 pathway regulates the immune response in lymphoid tissues as well as in target organs. Upon binding to either PD-L1 or PD-L2, PD-1 negatively regulates the antigen receptor signaling and immune activation by recruiting protein tyrosine phosphatase to dephosphorylate the downstream molecules involved in B cell receptor mediated signaling [21] and T cell activation [22]. PD-1-PD-L1 pathway plays integral role in developing central and peripheral immune tolerance by inhibiting proliferation and maturation of T lymphocytes [23, 24]. PD-1 is highly expressed in tumors and a large portion of Tumor-Infiltrating Lymphocytes (TILs), consisting of both CD4^+^  T_reg_ cells and CD8^+^ cells with resulting decreased production of cytokines [25]. The expression of PD-L1 is elevated, often responding to IFN-*γ* [26], in a variety of malignancies including gastroenterological cancers. PD-1-PD-L1 axis is exploited by tumors to inhibit tumor antigen-specific immunity and achieve tumor immunity escape [27–34]. Higher expression of PD-L1 in cancers is usually correlated to poorer prognosis [35–37]. In gastroenterological cancers, expression of PD-L1 is linked to higher *α*-fetoprotein level, blood vessel invasion, and overall poor prognosis of hepatocellular carcinoma [32, 35]. In addition, PD-L1 status is associated with visceral metastasis and more FOXP3^+^  T_reg_ cell infiltration in gastric cancers [27]. Similarly, in cholangiocarcinoma, PD-L1 expression is found in up to 30% patients and is linked to worse prognosis [30, 31]. Therefore, PD-L1 blockade can relieve tumor suppression, enhance tumor antigen-specific immunity, and improve prognosis. The antitumor activity of PD-1 blockade has been confirmed in both animal experiment [38] and clinical trials [39], where the tumor regression in response to PD-1 antibody treatment was observed in refractory solid cancers including colon, renal, and lung cancers and melanoma. It was observed that 22% of patients with PD-L1 positive metastatic adenocarcinoma of gastric or gastroesophageal junction showed overall response to Pembrolizumab, the humanized antibody to PD-1 receptor [40]. Cytotoxic T Lymphocyte Associated Antigen 4 (CTLA-4) is a coinhibitory molecule stored in intracellular vesicles of the naïve CD4^+^ and CD8^+^ T cells located in the lymph nodes, and it can be transported to the membrane of T cells and inhibit the activation of naïve T cells in the priming phase upon binding to its ligand B7 expressed on antigen-presenting cells (APC) [41]. CTLA4 expression is elevated by T cell activation status and an inflammatory environment for exerting brake on immune response [42]. Ipilimumab, a monoclonal antibody antagonizing CTLA-4, was approved by US Food and Drug Administration in 2011 to treat melanoma. Ipilimumab is undergoing clinical trials for other cancers including lung, bladder, and hormone-refractory prostate cancers.

Besides immune checkpoint blockade and immunosuppressive cytokine inactivation, therapeutic strategies targeting on enhancing activation of nature killer cells and macrophages, reversing the immune tolerogenic profile of tumor microenvironment, and ablating the immunosuppressive tumor-associated macrophages (TAMs) have been developed and evaluated. In addition, the low expression of the tumor antigen-derived peptide presented on major histocompatibility complex class I (MHC I) is a major reason for the limited clinical benefit of antigen-specific cancer immunotherapy [43]. Peptide intratumor injection has been shown to enhance tumor cell antigenicity for specific cytotoxic T lymphocyte activity and be effective in inhibiting tumor growth and prolonging survival time [44].

MicroRNAs (miRNAs) are a group of short (~ 22 nt), evolutionally conserved, single stranded noncoding RNA molecules that regulate expression of target genes by either cleaving mRNAs or destabilizing the translational system through interacting with sites of imperfect complementarity at 3' untranslated region (UTR) of target mRNAs [45]. With over a thousand of miRNAs present in higher eukaryotes and with each miRNA targeting on several genes, miRNAs can regulate the expressions of about 60% human protein-encoding genes [46]. miRNAs are involved in various biological processes including embryonic development [47], DNA repair [48], cell proliferation and senescence [49], differentiation [50], and apoptosis [51]. Dysregulation of miRNAs is associated with various diseases including Schizophrenia, obesity, alcoholism, and heart disease [52–54]. Notably, dysregulation of miRNA expression profiles is common in most malignancies, and the deregulation of miRNAs may lead to creation of favorable environment for the development of hallmarks of cancer [55]. The regulatory roles of miRNAs in metabolic and cellular pathways, especially those controlling cell proliferation, differentiation, apoptosis, and survival, are crucial to tumor initiation and progression.

Since miRNAs modulate the differentiation, activation, and mobilization of diverse immune cells and the complex cytokine network, miRNAs play vital roles in both innate and adaptive immunity. miRNAs regulates innate immune system by modulating the functions of its major players including natural killer cells (NK), microphage, and* γδ* T cells, as well as the production of inflammatory cytokines and chemokines [56]. NK cells achieve immune surveillance by cytotoxicity and type I Interferon-*α* (INF-*α*) activation. miR-27a has been demonstrated to negatively regulate NK cells by repressing the genes PRF1 and GZMB [57]. Also, miR-30c-1 is known to affect the activation of NK cells by regulating the expression of tumor necrosis factor *α* (TNF-*α*) and HMBOX1 [58]. For regulatory roles for macrophage, it was reported that miR-511-3p modulated the inflammatory activity of tumor-associated macrophage [59]. In addition, miR-125b has been shown to regulate the expression of inflammatory cytokines by repressing the expression of TNF-*α* in macrophage [60]. miR-19 was also reported to modulate NF-*κ*B mediated inflammation [61]. Adaptive immunity is characterized by specificity and memory of the immune response produced by the orchestrated interplay among T cell, B cell, dendritic cells, and complex network of inflammatory cytokines. miRNAs have been found to be involved in differentiation of B cells and the activation of T cells and dendritic cells [62]. miR-150 and miR-34 were found to inhibit B cells from developing from Pro-B to Pre-B stage through the downregulating c-MYB [63] and FOXP1 [64], respectively. Also, the expression of miR-155 was elevated in B cell precursors of lymphoblast leukemia, suggesting that miR-155 may cause development stalk and accumulation of pre-B cells by downregulating SHIP and C/EBP*β* [65]. In addition, miRNAs have been demonstrated to be involved in the regulation of T lymphocyte activation and the antigen-presenting ability of dendritic cells, which engage all other immune cells in the immune response. miR-135b was shown to negatively regulate Th2 lymphocyte regulator genes STAT6 and GATA3 [66]. Also, miR-140-5p, miR-409-3p, and miR-433-3p can regulate the tumor antigen recognition and cytotoxicity of CD8^+^ T lymphocytes and NK cells by regulating the expression of ULBP1, which is a ligand of NKG2D, an immunoreceptor found on T cells and NK cells [67]. Furthermore, Zheng et al. reported that the differentiation of FOXP3^+^CD4^+^ T regulatory cells and the tolerogenic property of dendritic cells could be enhanced by miR-23b through repressing the expression of NOTCH1 and the NF-*κ*B [67] (21406206). Moreover, microRNAs also regulate the immune checkpoint activity. For example, miR-155 overexpression in CD4^+^ T cells leads to decreased CTLA-4 levels and the subsequent activation of T cell immune response [68].

Since microRNAs are intricately involved in the modulation of activation of innate and adaptive immunity, in the regulation of inflammatory response and cytokine signaling, and in the molecular trafficking and cytokine crosstalk between the tumor and its microenvironment, miRNAs are promising targets for developing immunotherapy against gastroenterological cancers, for which the current targeted chemotherapy has not provided significant clinical benefit for overall response and survival. This review will focus on the regulatory roles of miRNAs on the immunity of digestive system and the antitumor immunity against gastroenterological cancers. For each type of cancers, miRNAs possessing the following functions will be featured: (1) directly modulating the activation of immune cells (macrophages, CD^+^4 or CD^+^8 T cells, T_regs_, NK cells, dendritic cells, etc.) and subsequently affecting growth and metastasis of cancers; (2) presenting a cytokine profile that shapes the immunosuppressive microenvironment of cancers; (3) directly impacting the cellular components of tumor microenvironment niche including the cells lodging in the tumor stroma that contributes to cancer immune evasion; (4) being implicated in the development of preneoplastic conditions (e.g., hepatitis B and C, liver lipid metabolism disorder, and steatohepatitis for liver cancers; Crohn's disease, ulcerative colitis, and other colonic inflammation for colorectal cancer); (5) being targeted by transcriptional factors (such as STAT3) that are involved in immunity and cancer immune evasion; (6) sensitizing the cytotoxicity of immunotherapeutic agents.

## 2. MicroRNAs' Immunoregulatory Ability in Hepatocellular Carcinoma (HCC)

The incidence of liver and intrahepatic bile duct cancers has been increasing in the past decade, with estimated 40,710 new cases and 28,920 deaths in 2017 in the United States [1]. Liver and intrahepatic bile duct cancers have been the fifth deadliest cancers in men and account for 4.8% of overall cancer-related cancers in both sexes [1]. Due to the increasing hepatic virus infections of both hepatitis B and C viruses and rising incidence of both alcoholic and nonalcoholic fatty liver disease (NAFLD), hepatocellular carcinoma has been the 5th most common cancer and the third leading cause of cancer-related deaths worldwide [69]. It is predicted that liver cancers would surpass breast, prostate, and colorectal cancers to become the third leading cause of cancer-related deaths worldwide by 2030 [70]. The low survival rate is attributed to the latent disease course, late diagnosis, metastasis, and recurrence. Sorafenib, the only FDA-approved drug for advanced HCC, improves overall survival by only 2.8 months [8]. Recent phase 3 clinical trials have shown that sorafenib did not improve the median recurrence-free survival as an adjuvant therapy after resection or ablation of HCC [71]. Therefore, it is imperative to develop novel treatment strategies employing therapeutic molecules that boost antitumor immunity for combating HCC.

### 2.1. MicroRNA Removes Immune Checkpoint Blockade Imposed by PD-1/PD-L1 Pathway

Immune checkpoint blockade using antibody antagonizing PD-1 achieved overall response for some gastroenterological cancers. However, due to intrinsically poor immunogenicity and suppressive desmoplastic tumor microenvironment, anti-PD-1/PD-L1 monotherapy had not shown significant therapeutic benefit [34, 39]. Therefore, the new strategy that combines anti-PD-1 antibody and PD-L1 expression knockout would further remove the immune blockage imposed by PD-1/PD-L1 pathway. Targeting PD-1 or PD-L1 genes, a number of microRNAs have been found to inhibit tumor growth, initiate PD-1 specific T lymphocyte apoptosis, and reverse chemoresistance by blocking PD-1 immune checkpoint [72, 73].

miR-34a has become a rising star of microRNA-based therapy since it targets on over 30 oncogenic genes across distinct cellular pathways, modulates immune response, and prevents cancer cells from achieving immune evasion. In addition to activating dendritic cell mediated innate immune response by repressing DAPK2/SP1 pathway in gastric cancer [74], miR-34a also increases tumor-infiltrating CD8 expression T lymphocytes and decreases CD8/PD1 expression T lymphocyte by directly targeting PD-L1 3'-untranslated region [75]. Clinical delivery of MRX34, a liposome formulated mimic of miR-34a and the first-in-human clinical trial of microRNA therapy, has been evaluated for treating advanced solid tumors including unresectable liver cancers and metastatic tumors with liver involvement. Some patients achieved prolonged confirmed partial response (PR) per Response Evaluation in Solid Tumors (REIST) or stable disease (SD).

### 2.2. MicroRNA Alters Immunosuppressive Cytokine Profile by Serving as an Effector of STAT3

Signal transducer and activator of transcription 3 (STAT3) is an important transcriptional factor for cell differentiation, proliferation, and death and is implicated in tumor induced immune suppression in hepatocellular carcinoma (HCC) [76]. STAT3 can inhibit antitumor activity of NK cells against HCC cells by suppressing the expression of NKG2D ligands and type 1 interferon (IFN) [76]. In addition, STAT3 signaling has been found to regulate both innate and adaptive immunity by increasing the expressions of growth factors and cytokines including TGF-*β*, VEGF, interleukin 6 (IL-6), and IL-10 [76–78], which collectively repress the host immune response and facilitate tumor immune evasion. Therefore, understanding the molecular mechanisms through which the effectors of STAT3 network contribute to STAT3-mediated immune suppression is valuable for developing therapeutic strategy for HCC. miR-146a has been shown to be a direct target of STAT3 and its expression is activated by binding of STAT3 to the promoter of miR-146a gene [76]. Inactivation of STAT3 leads to downregulation of miR-146a, which subsequently alters aforementioned STAT3-associated immunosuppressive cytokines profile and restores the function of NK cells and antitumor lymphocytes.

### 2.3. MicroRNAs Affect the Cytotoxicity of NK Cells

A number of microRNAs can quench the tumor response by suppressing the MHC class 1-related chain molecule A and B (MICA and MICB), which are expressed on tumor cells and are ligands of the natural killer (NK) cell activating receptor NKG2D [79]. Suppression of MICA and MICB decreases the susceptibility of tumor cells to the cytotoxicity of NK cells [80]. miR-20a, miR-93, and miR-106b are MICA/B-targeting microRNAs and are encoded by host genes miR-17-92 cluster and maintenance complex component 7 (MCM7) [79]. These MICA/B-targeting miRNAs have been found to contribute to immune response evasion, and the epigenetic downregulation of MICA/B-targeting miRNAs by histone deacetylase inhibitor sensitizes HCC cells to the cytolytic effect of NK cells [81].

### 2.4. MicroRNAs Regulate the Activation of Tumor-Associated Macrophages

Tumor-associated macrophages (TAMs) are infiltrating macrophage subpopulation differentiated from circulating monocytes induced by cytokines produced by type 2 T helper cells (Th2). Dual-specificity phosphatase (DUSP1) is a negative regulator of MAPKs and thus inhibits the production of cytokines including TNF-*α*, TGF-*β*, IL-1, and IL-6. miR-101 directly targets on DUSP1 and increases HCC growth and metastasis by suppressing the expression of DUSP1 and the resulting increase in proinflammatory cytokines [82].

miR-26a has been identified as a tumor suppressor that inhibits tumor growth and metastasis by downregulating its oncogenic targets [83]. For HCC, overexpression of miR-26a reduced the expression of macrophage colony-stimulating factor (M-CSF), decreased expression of chemokine (C-C motif) ligand (CCL)22, CCL17, and IL-10, and inhibited the macrophage infiltration in tumors [84].

### 2.5. MicroRNAs Are Implicated in Hepatitis B and C Infections, High Risk Factors of HCC

Hepatitis B and C can produce prolonged chronic inflammatory status and subsequent liver cirrhosis and eventually HCC. Since chronic infection of hepatitis B or C virus (HBV or HCV) is a high risk factor of HCC [85], microRNAs that affect the life cycle and infection of HBV or HCV also have profound effects on the tumorigenesis and progression of HCC.

miR-122 has been discovered to assist replication of hepatitis C virus RNA [86]. Based on this finding, a miR-122 inhibitor miravirsen was developed and achieved prolonged dramatically reduced viremia without dose-related toxicity in a phase 2a trial in HCV type I patients [87]. Even though anti-miR-122 drug has shown satisfactory clinical outcome without emergency of viral resistance, due to the integral roles of miR-122 in liver homeostasis and maintenance of hepatocytic phenotype, the long-term inhibition of miR-122 in patients with underlying liver pathological conditions needs more thorough clinical evaluations. In contrast, miR-122 expression has been found to be reduced in patients with HBV infection and negatively correlate to the virus load and necroinflammation of liver [88]. Transfection of miR-122 mimics inhibited virus production. miR-122 inhibits HBV replication by suppressing the expression of cyclin G_1_, which can negatively regulate p53-mediated inhibition of HBV transcription [88]. In addition, miR-122 has been found to suppress the interferon-stimulated response element (ISRE) mediated gene expression by enhancing methylation at gene promoter of suppressor of cytokine signaling 3 (SOCS3) and subsequently increasing expression of SOCS3 in liver cells [89]. Therefore, silencing miR-122 can improve response of liver cells to interferon-*α* in treatment against hepatic B or C, which are major causes of liver cirrhosis and cancer.

Besides miR-122, miR-185 interferes with the HCV life cycle by targeting several genes encoding critical proteins for entry, replication, and assembly of HCV infection. In addition, through inhibiting the lipid accumulation and other immunometabolic modulation, miR-130b and miR-185 inhibit the infection of hepatitis C virus by reinforcing the antiviral activity of 25-hydroxycholesterol (25-HC), an oxysterol secreted by interferon-stimulated macrophages and dendritic cells [91].

### 2.6. miR-122 Is a Liver-Specific MicroRNA That Plays Vital Roles in Liver Homeostasis and Immunity

The implication of miR-122 in the infection of HBV and HCV discussed above provides an example of how miR-122 is involved in liver disease. miR-122 is the most abundant liver-specific microRNA, constituting about 70% of overall microRNAs in liver. It is conserved in all vertebrates, indicating its crucial roles in the liver [115, 116]. miR-122 plays important roles in lipid metabolism, iron homeostasis, and maintenance of hepatocyte differentiation by regulating a large number of genes involved in various hepatic functions and repressing nonhepatic genes [117, 118]. The expression of miR-122 is downregulated in human HCC patients [119], and the deletion of miR-122 in hepatocytes leads to progressive development of stages of liver cancer: steatohepatitis, inflammation, hepatocyte regeneration, fibrosis, cirrhosis, and primary and metastatic HCC [120]. The loss of miR-122 has been associated with metastasis and poor prognosis of HCCs [121], whereas the restoration of ectopic miR-122 suppresses cell replication and invasion, inhibits angiogenesis, and sensitizes the liver cancer cells to sorafenib. miR-122 has been found to contribute to the liver disease outcome by modulating both innate and adaptive immunity. Mice with miR-122 knockout develops chronic inflammation that progresses to HCC since miR-122 deletion leads to upregulation of chemokine (C-C motif) ligand 2 (CCL2) in liver [90]. CCL2 recruits CCR2^+^CD11b^high^Gr1^+^ immune cells to the liver, where these cells produce proinflammatory cytokines and subsequently cause hepatitis and progressively HCC [122].

In addition, miR-122 is significantly downregulated in primary biliary cirrhosis (PBC), which is an autoimmune disease that causes destruction and cirrhosis of intrahepatic bile duct [123]. The deregulation of miR-122 in PBC suggests that miR-122 either mediates the intrahepatic bile duct cell damage or modulates the autoimmune reaction process.

## 3. The Implications of MicroRNAs in Immunotherapy for Pancreatic Cancer

It is estimated that there will be 53,670 new pancreatic cancer cases and 43,096 death cases in the United States in 2017 [1]; both will be higher than the cases in 2016. Although pancreatic cancer is the eleventh most cancer among men and ninth most cancer among women, it is the fourth leading cause of cancer death of both sexes, accounting for 7% of overall death caused by cancers [1]. Limited progress has been made for improving the outcome of pancreatic cancer, with its 5-year survival rate modestly increased from 2.5% in 1975-1977 to 8.5% in 2006-2012 [124]. Due to the rapid development of therapies for other cancers, pancreatic cancer is projected to become the second leading cause of cancer death by 2030 [70]. The early systemic metastasis is the primary reason for the grooming prognosis of this disease.

### 3.1. MicroRNAs Transferred by Tumor-Derived Exosomes Engage in Immune Regulation

Tumor-derived exosome, a secreted membrane vesicle produced from inward budding of endosomal membrane, is generally considered as a promising source of tumor vaccine since it contains abundant immune regulatory proteins including MHCI [125], MHCII [126], and heat shock protein 70 [127] and various tumor rejection antigens including gp96, Her2, and tyrosinase related protein (TRP) [128]. Tumor-derived exosome presents tumor specific antigens to dendritic cells and induced potent CD8^+^-dependent antitumor [128]. Due to the antitumor immune activation capacity, nanoscale size (30-100nM), and chemical stability, exosome has been targeted and exploited to develop novel cancer immunotherapeutic vaccine, which progressed to the clinical trials [129]. Since tumor-derived exosome contains various cytosolic components of donor cells, it can profoundly modify the biological behaviors including immune responses of recipient immune cells (macrophage, dendritic cells, NK cells, T lymphocyte, etc.) in proximity as well as at distance sites by transferring signaling molecules, receptors, enzymes, and gene expression regulatory molecules including microRNAs. Exosome was found to mediate the microRNA transfer and thus the exosome was proposed to be a novel mechanism of gene transfer between cells [130]. Since exosomes can be secreted by a myriad of tumors and immune cells including dendritic cells, macrophage, B cells, cytotoxic T lymphocytes, fibroblasts, platelets, mastocytes, and tumor cells [128, 131], microRNAs transferred via exosome can effectively affect the tumor antigen-specific immune response of the immune cells and facilitate the immune tolerance of tumor cells. It was reported that miR-203 was present in pancreatic cancer cell derived exosomes, and miR-203 suppressed the expression of toll-like receptor 4 (TLR4) resulting in the decreased levels of tumor necrosis factor-*α* (TNF-*α*) and interleukin-12 (IL-12) in pancreatic cancer cell line Panc-1 [92]. Since TNF-*α* and IL-12 both play critical roles in maturation of immune cells, enhancing antigen presentation of APCs, and augmenting cell immunity, miR-203 may modulate TLR-mediated immune response and facilitate immune escape of pancreatic cancer cells. The engagement of microRNAs in the immune regulation of pancreatic cancer was further consolidated by the discovery that the pancreatic cancer derived exosome with depleted microRNAs by ultrafiltration displayed stronger potency of activating dendritic cells and cytokine induced killer cells to exert cytotoxicity against pancreatic cancer cells [132].

### 3.2. MicroRNAs Disarm PD-1/PD-L1 Immune Blockade Pathway

Like in HCC, microRNAs targeting PD-1/PD-L1 pathway can suppress pancreatic cancer progression by activating anticancer immunity. Among these microRNAs, miR-142-5p was found to downregulate the expression of PD-L1 by directly binding 3' UTR of PD-L1 mRNA, and the overexpression of miR-142-5p increases CD4+ and CD8+ T lymphocytes and concomitantly decreases PD-1+ T lymphocytes [93]. Therefore, miR-142-5p is promising in developing microRNA-based therapy targeting PD-1/PD-L pathway.

### 3.3. MicroRNAs Regulate the Functions of Immune Cells and Lymphatic Vessel Formation

MicroRNAs have been found to directly regulate the maturation, recruitment, and activation of macrophages and NK cells in the microenvironment of pancreatic cancers. miR-454 directly targets and downregulates stromal cell derived factor-1 (SDF-1), which is a target of hypoxia-induced factor-1 (HIF-1). Since macrophages are recruited to tumor tissues by its expression of CXCR4 in response to SDF-1, miR-454 was found to inhibit the recruitment of bone marrow-derived macrophages to pancreatic ductal adenocarcinoma (PDAC) cells by downregulating SDF-1 [94]. In addition, miR-146a expression level is elevated in dendritic cells treated by conditioned medium of pancreatic cancer cells, which leads to impaired differentiation and antigen presentation function of dendritic cells [133]. Therefore, miR-146a is implicated in the maturation and antigen presentation of dendritic cells in response to the invasion of pancreatic cancer cells.

miR-206 has been identified as a negative regulator of proinflammatory factors including the chemokines (C-X-C motif) ligand 1 and (C-C motif) ligand 2, Interleukin-8, and the granulocyte macrophage colony-stimulating factor in pancreatic adenocarcinoma. It also abolishes the expression of prolymphangiogenic factor, vascular-endothelial growth factor C, thus inhibiting blood and lymphatic vessel formation and suppressing tumor progression [95].

### 3.4. MicroRNAs Regulate the Inflammation of Pancreatitis, a High Risk Factor of Pancreatic Cancer

Acute pancreatitis (AP) is a sterile inflammation in pancreas with severity ranging from mild to high mortality despite aggressive medical intervention. AP is a risk factor of pancreatic cancer. MicroRNAs were aberrantly expressed in acute pancreatitis and can serve as biomarkers for diagnosis and prognosis [134, 135]. miR-9, produced by bone marrow-derived mesenchymal stem cells (BMSCs), negatively regulates the inflammatory response induced by lipopolysaccharide (LPS). It was reported that miR-9-modified BMSCs (pri-miR-9-BMSCs) significantly decreased release of inflammatory factors and reduced pancreatic injury, indicating that miR-9 may play an anti-inflammatory role in the pathogenesis of AP and a promising candidate target for microRNA-based treatment of AP [96].

miR-216a is a highly expressed miRNA in pancreas. miR-216a is found to repress the expression of PTEN, Smad7, pAkt, and TGF-*β* receptor 1 and, thus, it is implicated in the pathogenesis of AP. Inhibition of miR-216a expression by TGF-*β* inhibitor significantly decreased serum amylase, TNF-*α*, IL6, and TGF- *β* and alleviated histopathological changes of pancreas [97].

Chronic pancreatitis (CP) is defined as “a pathologic fibro-inflammatory syndrome of the pancreas in individuals who develop persistent pathologic responses to parenchymal injury or stress.” [136]. CP is characterized by chronic inflammation of the pancreas and the resulting progressive pancreatic endocrine and exocrine dysfunction [137] and widely accepted to be a strong risk factor of pancreatic cancer [138]. The microRNA that is known to be involved in CP is miR-146a, which suppressed the production of proinflammatory factors including IL-1*β*, IL-6, and TNF-*α* and is involved in innate immunity and inflammatory response pathways [98]. A common G/C SNP polymorphism rs2910164, which is located in the crucial seed sequence of the mir-146a, is found to affect the expression of mature miR-146a and is correlated to the increased chronic pancreatitis risk [139].

## 4. Immunoregulatory Roles of miRNAs in Colorectal Cancer (CRC)

The estimated number of new cases for colorectal cancers in 2017 for the United States is 135,430. The only cancers with higher rates of new cases are lung and bronchus, breast, and prostate. In fact, it is estimated that 50,260 people will die from CRC in the United States in 2017, which is only behind lung and bronchus cancers [1].

### 4.1. miRNAs Regulate the Regulatory T Lymphocytes

In order to display the relevance that microRNA has in CRC, Cobb et al. performed a Dicer KO in Treg cells. This study displayed that Dicer was a requirement for Treg cell development. Without Dicer, Treg cell numbers are diminished [140]. Moreover, a previous Dicer KO study has shown that cytotoxic T lymphocytes (CTLs) have increased ability to lyse CRC cells [99, 101]. This is thought to occur through the inactivation of miR-222/339, which typically downregulates ICAM-I [99]. However, due to the essential role of Dicer in RNA regulation, targeting it in patients would lead to unwanted side effects. Therefore, future studies should look at using anti-miRs to directly knock down miR-222/339.

Another method for regulating T cells with microRNAs in CRC is with miR-21. According to a recent study by Mima et al., miR-21 is inversely associated with CD3^+^ and CD45RO^+^ T cells. The proposed mechanism for this is by targeting PDCD4, which is a known IL10 repressor [100]. The correlation was determined by looking at a cohort of 538 cases of CRC. This is significant because it shows the ability for miR-21 therapy to improve the immunity against CRC. Specifically, CD3^+^ cells are known for their ability to activate CD3^+^ and CD8^+^ cells, which will increase the likelihood of antitumor activities in these cell types [141]. In addition, CD45RO^+^ T cells are a population that has been associated with increased TNM stage but still needs further investigation [142].

### 4.2. miRNAs Modulate the Function of MDSCs

Cancer cells and their co-opted microenvironments often take a multipronged approach to evade immune responses. For this reason, it is important for scientists to also look at multipronged approaches in order to improve immune responses. Myeloid-derived (immune) suppressor cells (MDSCs) are often a key player in creating the microenvironment. These cells are able to control CRC and immune cell development. Typically, MDSCs inhibit the growth of antigen-specific CD4^+^ and CD8^+^ cells [143]. However, we are able to modify the function of MDSCs with microRNA. The miR-17-92 cluster contains miR-17-5p and miR-20a. These microRNAs are both beneficial and harmful to CRC development. Unfortunately, they are found to promote CRC development by decreasing the burden of reactive oxygen species [101]. However, they are also able to inhibit the immunosuppressive action of MDSCs [102]. Thus, their usefulness for treating cancer still needs further exploration. Another mechanism that modulates MDSC action is with miR-494. miR-494 expression inhibits PTEN while simultaneously activating AKT. This leads to an increased number of procancer MDSCs [144].

### 4.3. miRNAs Modulate the Pathogenesis of Inflammatory Diseases of Colon

The role of miRs in chronic inflammatory disorders is also of key importance due to the ability of these disorders to induce CRC [101]. Some of the strongest associations have been with ulcerative colitis, Crohn's disease, and inflammatory bowel disease [145]. In ulcerative colitis, overexpression of STAT3 is common and leads to progression from ulcerative colitis to CRC. miR-124 targets STAT3 and is downregulated in these patients [103]. miR replacement therapy could be a viable option for these patients to decrease the risk of developing CRC. In patients with Crohn's disease, miR-19b has been identified as an anti-inflammatory molecule that has potential to decrease the tumor-promoting capability of Crohn's disease. miR-19b does this by modulating the expression of cytokine suppressors [146]. Moreover, miR-210 has a similar ability to decrease inflammation in inflammatory bowel disease by targeting HIF-alpha [104]. In addition, miR-511-3p, carried on the gene of the macrophage mannose receptor CD206 (mrc-1), is expressed by macrophage and dendritic cells. miR-511-3p targets toll-like receptor 4 (tlr-4) and reduces the production of proinflammatory cytokines in response to microbial stimulus. Therefore, miR-511-3p regulates the intestinal inflammation by regulating toll-like receptor 4 [147].

### 4.4. miRNAs Regulate the Recruitment and Activation of Macrophage and Neutrophils

miR-484 has been found to inhibit CD137L and thus have significant anticancer properties. CD137L has two characteristics that make it a procancer protein. First, CD137L induces cell viability via the PI3K and mTOR cell pathways. Secondly, CD137L induces IL-8 production which is used to recruit macrophages and neutrophils into the procancer microenvironment. If this is successful, the procancer macrophages and neutrophils then assist with tumor invasiveness. In microsatellite instable CRC, miR-484 plays a key role in regulating IL-8 secretion [105]. IL-8 is dangerous for the tumor microenvironment because of its proliferative effects and ability to promote tumor-associated immune cells such as macrophages and neutrophils into the microenvironment [148].

Procancer neutrophil function has been elucidated more in recent years. Although not all neutrophilic functions promote cancer, one example of cancer induction is shown by their ability to inhibit the immune system [149]. Along with other functions they have also been found to help tumors induce angiogenesis [150]. The role of tumor-associated macrophages in cancer has been more clearly elucidated, stimulating malignancy via metastasis, angiogenesis, and immunosuppression [151]. Fortunately, miR-484 and miR-19a have been found to inhibit CD137L and thereby improve the cancer microenvironment [152, 153].

### 4.5. miRNAs Regulate the Expression of PD-L1

MicroRNAs are also able to improve the immune response to CRC through targeting PD-L1. PD-L1 has been popularized recently due to its ability to reduce the viability of T cells [154]. It has also been discovered that miR-142-5p targets PD-L1 [93]. Moreover, miR-20b, 21, and -130b are overexpressed in CRC and target phosphatase and tensin homolog (PTEN), leading to PD-L1 overexpression [101, 106]. These events in turn block the PD-L1/PD-1 signaling pathway, allowing T cells to survive in the tumor microenvironment, thus improving anticancer immunity.

## 5. Immunoregulatory Roles of MicroRNAs in Gastric Cancer (GC)

Although the rate of gastric cancer has been decreasing, there are still around 22,220 patients diagnosed annually in the United States with approximately 10,990 annual deaths [155]. Moreover, the rate of noncardia gastric cancer for whites aged 25 to 39 in the United States increased between 1977 and 2006, showing that it is still a disease that needs to be studied further [156]. MicroRNA has also been discovered to modulate the anticancer immune response in GC, although in a somewhat convoluted manner. It is understood that the downregulation of E2F-1 in dendritic cells successfully inhibits the immune response. E2F-1 controls the activity of P53 and also regulates cell activity including proliferation [157]. Moreover, death-associated protein kinase 2 (DAPK2) along with the transcription factor specificity 1 (SP1) is able to inhibit the activity of E2F-1. This also leads to an inhibition in the activity of dendritic cells in the immune response. However, we can counteract this procancer activity by inhibiting the expression of DAPK2/SP1 using miR-34a [74].

## 6. MicroRNAs' Immunoregulatory Ability in Gallbladder Cancer

Gallbladder cancer (GBC) is a difficult to diagnose gastroenterological cancer. Detection in early stages is limited due to a poor understanding of the mechanisms involved in the development of cancerous hyperplasias [158]. The diagnosis of GBC is typically seen in advanced and late stage development. Unfortunately, this accounts for a 5-year survival rate of only 20-40% [159]. The available literature on GBC investigates methodologies that would potentially allow for more progressive detection. Abnormalities in cytokine profiles in the cellular microenvironment have long been a known contributor to oncogenesis. Current advancements and continued research allow for advanced genetic screening capabilities. With the capacity to screen for a range of miRNA and lncRNA, early diagnosis seems more promising.

The gallbladder is the bile producing organ of the body. These biliary secretions are channeled through ducts from the gallbladder to the liver. In a pancreaticobiliary maljunction (PBM) the bile ducts themselves are conjoined in such a way that allows for reflux of biliary secretions [107]. The presence of a PBM presents an increased risk factor for the development of GBC. This abnormal buildup of bile causes lecithin to convert endogenously to lysolecithin, which has been shown to induce chronic inflammation of the biliary epithelial cells [160]. The inflammatory response to lysolecithin disrupts the extracellular microenvironment causing an influx in inflammatory cytokines [161].

### 6.1. Cytokines and GBC

Cytokine expression profiles of healthy gallbladder cells in mice were shown to express mRNA for TNF-*α* along with RANTES and macrophage inflammatory protein 2 (MIP-20). Once these cells were treated with lipopolysaccharides, the expression profiles changed to exhibit an increase in mRNA for monocyte chemoattractant protein-1 (MCP-1), Interleukin-6 (IL-6), and IL-1b [162]. With immunotherapeutic treatments these abnormal cytokines could be used to aggregate genetically enhanced immune cells to the site of carcinoma. However, such an inflammatory response can thus lead to mutations and overexpression of cell-cycle associated proteins such as p53 and MUCI [163]. The implications of p53 mutations has been shown to potentiate atypical hyperplasia, thus resulting in lesions and subsequent malignancy [163]. MUC1 is a mucin class glycoprotein produced by epithelial cells and involved in cell signaling and adhesion. Left unchecked, this overexpression thus potentiates the formation of cancerous cells in the lining of the biliary tract. This protein has been found in increased, lowered, and erratic expression in tumor cells related to GBC [163]. Expression profiles of MUCI and MUC5AC showed predictable patterns which accounted for metastasis of tumor cells. H. Kono et al. demonstrated that, along with P53, K-ras mutations have also been studied as biomarkers in GBC with PBM [160], with K-ras being a well understood and studied prooncogene [164]. In addition, miR-133a-3p was reported to inhibit the gallbladder carcinoma by directly targeting on recombination signal-binding protein J*κ* (RBPJ) [109]. RBPJ is a key downstream transcription factor in the Notch signaling pathway that regulates the differentiation of T cell lineage from common lymphoid precursor.

### 6.2. miRNA and lncRNA in GBC Prognosis

Other potential biomarkers associated with GBC include a range of miRNA and lncRNA (long noncoding RNA). lncRNA CCAT1 (colon cancer-associated transcript-1) has been shown to promote GBC development through downregulation of miRNA 218-5p. This regulatory ability is associated with the direct binding of miRNA 218-5p to CCAT1 [107]. This class of lncRNA is categorized as ceRNA (competitive endogenous RNA) which acts as molecular ‘sponges' through endogenous miRNA binding sites. This mechanism could be a factor in the downregulation of tumor suppressing miRNA seen in miRNA profiles in GBC [158]. Alternately, overexpression of miRNA 155 has also been shown to increase the malignancy of late neoplastic conditions in GBC. This miRNA shows promise as a potential for detection in patients with symptoms associated with GBC. miRNA 155 elevation was also associated with an increase in metastasis to the lymph node [160]. In a miRNA study performed by P. Letelier et al. in human GBC cell lines, it was determined that miR-1 and miR-145 possess antitumor properties. These miRNAs along with miR-143 and miR-133 directly targeted signaling pathways associated with cell motility and adhesion, thus having implications in malignancy and carcinoma [110].

## 7. Immunoregulatory Ability of miRNAs in Esophageal Cancer (EC)

In esophageal cancer (EC), there can be several differing morphologies characterized by the cells that are affected. In ESCC the epithelial tissue lining the esophageal tract becomes cancerous [165]. Another manifestation of esophageal carcinoma is known as esophageal adenocarcinoma (EAC) which effects the glandular epithelium [166]. The manifestation of these carcinomas has been shown to increase with the prominence of a condition known as Barrett's esophagus (BE) where the cells of the squamous epithelium are replaced by glandular epithelium [165]. When analysis of both transcriptomes was performed, notable differences arose between the two subtypes of esophageal cancers. Esophageal squamous cell carcinoma (ESCC) is a high risk and difficult to detect carcinoma. Not accounting for the western world, ESCC is the one of the most diagnosed cancers [167]. Tobacco smoking and chronic alcohol consumption have been shown as risk factors for ESCC, with a high prevalence in Asian countries [165]. With a 5-year survival rate of 4.5%, early detection and prognosis are prudent for successful remission. Nonetheless, recurrence of ESCC is common and met with low rates of survival. Given these grim statistics, development of alternative detection and therapeutic methods is crucial.

### 7.1. lncRNA in EC

W. Wu et al. set out to characterize potential noncoding DNA and its implications on EAC. Through hypomethylation of DNA overexpression of otherwise noncoding regions can occur. This lack of methylation could induce genomic instability and downstream activation of oncogenes [14]. In EAC it was found that the lncRNA AFAP1-AS1 was subsequently overexpressed due to hypomethylated DNA regions in the transcriptome of EAC cells. The overexpression of AFAP1-AS1 was shown in vitro to induce cell proliferation and tumorigenic growth in EAC cell lines [166]. This invasiveness and tumorigenic growth was reversed with treatment using AFAP1-AS1-specific siRNA, showing promise for siRNA-targeted therapies. The researchers also postulated that AFAP1-AS1 is a potential biomarker for the detection of EAC. The exact function of AFAP1-AS1 is unknown and further studies must be performed. lncRNA MALAT1 has also been shown to induce and promote tumorigenesis in ESCC. In a study to elucidate the potential for MALAT1 miRNA therapies X. Wang et al. demonstrated in vivo suppression of ESCC growth by using miR-101 and miR-217. These mi-RNAs were shown to target MALAT1 consequentially silencing its tumor-promoting action [167].

### 7.2. miRNA in EC

Potential for noncoding RNA biomarkers for esophageal cancer (EC) includes a wide range of dysregulated miRNAs. A study performed by Shang-guo Liu et al. set out to establish a baseline of miRNA expression profiles in human EC. To achieve this, researchers took EC patient cells and analyzed them using miRNA chip technology; these results were then further verified using RT-qPCR methods for comparison to noncancerous cells. This research identified 59 upregulated miRNAs along with 9 downregulated miRNAs [168]. According to R. B. Koumangoye et al., miR-31 was shown to suppress tumor oncogenesis when SOX4, EZH2, and HDAC3 genes are downregulated. However, in ESCC these genes are typically expressed and were shown to cooperatively downregulate the effectiveness of miR-31* in vitro* [169]. Measuring levels of miR-31 could provide a potential biomarker in GSCC detection. More notably, miR-31 directly regulates Stk40 and activates the STK40-NF-*κ*Β-controlled inflammatory pathway in esophageal cancer. Therefore, the anti-miR-31 can repress inflammation and neoplasm of esophageal cancer [111]. Invasion and metastasis is controlled by a host of proteins responsible for motility and cytoskeletal construction and distribution. FSCN1 and MMP14 are proteins that facilitate tumor progression and formation of a suitable tumor microenvironment. N. Akanuma et al. performed a study correlating the anticancer effects of miR-133a through its direct modulation of these two proteins [170]. This shows promise for miR-133a as a tumor suppressor in ESCC. Further studies have identified a host of well-defined miRNAs that elicit a notable and prominent promotion of migration and invasiveness of GC cells. miR-92 was found to be highly expressed in ESCC and to modulate the migration and invasion of ESCC cells through repressing the tumor suppressor CDH1 gene [171]. Y. Tian et al. studied the effects of miRNA-10b along with elucidating its target gene KLF4. This study showed that with overexpression of miR-10b came a direct underexpression of the tumor suppressor KLF-4. Along with this study, the researchers also performed a literature review showing miR-10b expression being downregulated in 95% of human EC tissues [172]. This direct correlation of miR-10b and tumorigenesis suggests potential as a generalized biomarker for EC detection. ESCC senescence and apoptosis have also been shown to be mediated by the expression of miRNA. miR-34a was shown to act as a p53-dependant tumor suppressor [173]. The expression of miR-34a was shown to be affected by DNA damaging agents traditionally used in chemotherapy. This expression was notably downregulated by the mutant p53 variant. Through the patient specific regulation of the wild type p53/p21 pathway miR-34a can be used as a diagnostic marker for therapeutic effectiveness in p53 mutant ESCC. In addition, miR-34a expression can be elevated by NF-*κ*B, a central regulator of inflammation [112].

### 7.3. The Implication of Exosomes Transporting miRNAs in EC

Secretion of exosomes is a well-known method of cellular communication and excretion. Small noncoding RNA molecules such as miRNA can also be secreted in exosomes and circulate in the blood or other extracellular matrix [114]. Exosome secretion adds to the plethora of intercellular communicatory systems that play a large role in mediating the cellular microenvironment. Through the use of Transmission Electron Microscopy and western blot analysis, the exosomal protein CD63 was confirmed in serum exosome isolate purified by Y. Tanaka et al. showing a reliable analysis of serum exosomes. Exosomal miR-21 was confirmed using a bioanalyzer in serum samples [114]. This research showed that the transmission of extracellular miRNA was indeed possible and could be transmitted intercellularly. This study also elucidated miR-21 as a potential cancer promoting miRNA which was determined through previous studies to increase proliferation and oncogenesis in vitro [174].

### 7.4. Immunosuppression in GSCC

CD4 and CD25 regulatory T cell (Treg) expression has been shown to increase in association with both gastric and esophageal cancer (EC) progression. Treg cells, which have been shown to inhibit immune responses otherwise mediated by T cells, have been noted in higher abundance with proximity to cancerous lesions. K. Kono et al. looked at the prevalence of Treg cells in PBMCs (Peripheral Blood Mononuclear Cells) in patients with ranging progression of both gastric and esophageal cancers. Through flow cytometry, RT-PCR and intracellular cytokine assays, the researchers were successful in separating Treg cells by unique markers such as CD45RO and CTLA-4 [175]. The proportional analysis also revealed a correlation between cancer stage and Treg prevalence. A speculative outlook on why this happens could be explained by the expression of chemokine CCL20, secreted by macrophages at the site of tumorigenesis. This chemokine has been shown to facilitate Treg migration [176]. Otherwise, the exact mechanism of action that allows for such an increase in Treg cells is currently unknown. Response to traditional inflammatory factors could also play a large role. A well-documented and studied protein pathway for inflammation induced cancer is NF-*κ*B and STAT3 [165]. These proteins have been shown to be expressed when induction of an inflammatory response occurs. Once induced, these proteins potentiate downstream cascades, activating a spectrum of oncogenes such as miR-21 and IL-6 [165].

Along the same lines of intracellular immunosuppression, myeloid-derived suppressor cells (MDSCs) have also been studied as a potential antagonist in immunotherapy. In a study performed on EC patients' PBMCs, R. F. Gabitass et al. demonstrated that elevated numbers of MDSCs were also associated with an increase of Treg cells in EC [177]. MDSCs impose immunosuppression through impairment of T cell differentiation. This overexpression was strongly correlated with elevated levels of the Th2 cytokine IL-13. By utilizing IL-13, Treg, and MDSC detection this study sheds light on these potential prognostic biomarkers EC detection. From an immunotherapeutic perspective, the abundance of MDSC and Treg cells presents a challenge in therapies which rely on the successful implantation of genetically altered T cells [177].

## 8. Immune Checkpoint Blockade and miRNA Therapy

**Figure 1:**
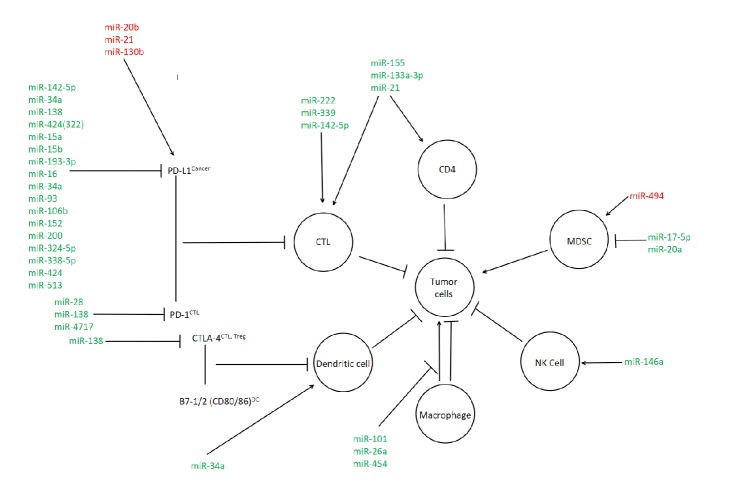
The miRNA modulation of cellular immunity in gastrointestinal cancers. Red denotes procancer miRNA interactions while green denotes anticancer interactions.

With the known antioncogenic and microenvironment altering properties of miRNA, the potential positive effects on the immune mediated response are of great interest. A critical adaptation of cancerous cells is their ability to imitate otherwise healthy cells and avoid immune detection [178]. In current literature, immune checkpoint blockade in cancer cells has been shown to elicit antitumor effects in late stage cancer development [179]. The widely studied and targeted immune checkpoint proteins are CTLA-4, PD-1, and PD-L1 [73, 180, 181]. PD-1 and PD-L1, also known as programmed death receptor and ligand, are of particular interest as the PD-L1 ligand is expressed on tumor cells, thus evading the imminent immune response [182]. The modulation of CTLA-4 is not as researched as the PD pathways; however miR-138 was shown to elicit an effect [183]. A major setback that has been seen when immune checkpoint antibodies are used in combinatorial therapy on multiple checkpoint pathways is risk of toxicity [183]. This is due to the nature of the immune checkpoint receptors being present on most cells. However, the use of miRNA to induce immune checkpoint blockade by selectively upregulating or downregulating miRNA expression for specific immune checkpoint proteins could prove to be a useful tool in our antioncogenic arsenal.  Figure 1 presents possible miRNA targets that are involved in the modulation of PD-L1, PD-1, and CTLA-4; this figure is modified and adapted from Q. Wang et al. [182].

## 9. Challenges and Future Study

Gastroenterological cancers have been considered to be poorly immunogenic, and, along with enhanced immune checkpoint inhibition, cancer cells can escape from the recognition and clearance of immune system. Pembrolizumab, a monoclonal antibody antagonizing PD-1 receptor, and Ipilimumab, the monoclonal antibody binding CTLA-4, have achieved some clinical success [40, 42]. However, due to poor immunogenicity and desmoplastic tumor microenvironment, mono-immunotherapy using antibody for achieving immune checkpoint blockade has not shown therapeutic benefit. The combination therapy combining traditional monoclonal antibody-based immunotherapy and the novel miRNA-based immunotherapy is expected to achieve more effective and sustained therapeutic effect. Liposome formulated mimic of miR-34a entered the first-in-human clinical trial of microRNA therapy and obtained favorable clinical results with prolonged partial response. In addition, since STAT3 has been found to play crucial roles in magnitude of tumor mediated immunosuppression and immune escape in tumor microenvironment [76, 78], miRNA-based immunotherapy targeting on STAT3 should be aggressively pursued.

The extensive involvement of miRNAs in modulation of tumor-associated immune cells, immune checkpoint blockage, and maintenance of immunosuppressive status of tumor microenvironment presents miRNAs as promising therapeutic agents or molecular targets for developing novel immunotherapy for awakening or augmenting antitumor immunity. The miRNAs, the target genes, and the immunoregulatory roles of these miRNAs in some gastroenterological cancers are summarized in Table 1. However, since a miRNA can regulate a number of target genes that may have fundamentally different and even contradictory effects on regulating immune response to tumors and the gene expression regulatory capacity of miRNAs and the biological roles of their target genes may be vastly different in various tissues and pathophysiological status, the actual net immunoregulatory effect of a miRNA has to be evaluated within a holistic and evolving immunomodulation network. Some microRNAs are known to positively regulate the functions of immune cells and enhance antitumor activity; however, augmenting immune response alone does not warrant the tumor suppressing effect of the microRNAs. The roles of miR-155 in antitumor immunity present a good example. miR-155 expression in T cells is required for maintaining the number of IFN*γ*-expressing CD4^+^ and CD8^+^ T cells and suppressing tumor growth [108, 184]. miR-155 also modulates the innate immunity by regulating IFN*γ* production in NK cells [185]. In addition, miR-155 in liver macrophage Kupffer cells plays an important role for normal antigen-presenting function and SOCS1/JAK/STAT inflammatory pathways of Kupffer cells [113]. Therefore, miR-155 can serve as a regulator of tumor immunity that enhances the tumor antigen recognition, surveillance, and clearance. However, miR-155 is highly expressed in multiple solid tumors and is commonly associated with more aggressive phenotype. miR-155 was reported to promote invasiveness of pancreatic cancer cells, through regulating suppressor of cytokine signaling 1 (SOCS1) and P-signal transducer and activator of transcription-3 (STAT3) [186], and promote the hepatocellular carcinoma progression by targeting PTEN [187]. In addition, expression of miR-155 in tumor tissue at a high level is positively correlated with lymph node metastasis, as well as poorer overall and disease-free survival in colorectal cancer patients [188]. Similarly, miR-155 expression is a biomarker indicating poor prognosis for gallbladder cancer [189]. Therefore, the precise delivery of microRNA mimics or inhibitors to specific cell types in tumor tissues or immune system is the key to the success of microRNA-based immunotherapy.

Differential expression level of the same miRNA in different tissues and cell types further complicates the prediction of the role of the miRNA in antitumor immunity. In addition, it is known that he expression of miRNAs is deregulated in tumors compared with that in normal tissues. However, the normal baseline level of miRNAs varies dramatically in different individuals. It is not uncommon that literature reports inconsistent or even conflicting discoveries regarding the expression level of miRNAs. Therefore, a challenge for better understanding the immunoregulatory roles of miRNAs and developing miRNAs-based immunotherapy is establishing a set of endogenous miRNA controls for normalization.

Another challenge lies in the difficulty of identifying miRNA candidates for treating a tumor due to the heterogeneity of miRNAs in tumor tissues. Inflammation and hypoxia in tumor microenvironment cause complex and dynamic heterogeneity in miRNA expression profile [190, 191]; therefore, multiple-point biopsy and temporal monitoring of expression of mRNAs are critical to the constructing of a more meaningful regulatory network of miRNAs and the identifying of miRNA candidate (usually the common regulatory miRNA) for developing immunotherapy for that particular cancer.

Safe, efficient, stable, and specific delivery of miRNAs to tumor neoplasm and microenvironment has been a major challenge for any RNA-based therapy [192]. Cationic polymers or viral vectors are efficient delivery vehicles; however, the systemic toxicity and immunogenicity cause significant side effects and limit the transition from bench to bedside. Recently, the delivery system that loads miRNA mimics or anti-miRNAs to nanoparticles conjugated with targeting antibodies/peptides displayed efficient targeting and achieved high cellular uptake and bioavailability [193].

Off-target effect is another challenge for developing miRNA-based immunotherapy. Since many miRNAs belonging to the same family have very conserved seed sequence and most anti-miRs contain perfect complementary sequences to the seed sequence, anti-miRs are not able to distinguish the miRNAs of the same family and may produce off-target effect [192]. A novel strategy addressing off-target effect targets on miRNAs at their precursor stage. Since the precursors of miRNAs contain secondary structure that is required for recruiting enzymes and the miRNA precursors contain sequences that are not found in seed sequences, anti-miRs can be designed to specifically bind to nonseed sequences on the miRNA precursors, which may disrupt the hairpin and affect the RNA processing by Drosha-DGCR8 complex [192]. Therefore, targeting secondary structure scaffold sequence but not seed sequence may help avoid off-target effects. Continued effort on assessing the effectiveness and off-target effect of this novel strategy is important for developing a safe miRNA-based immunotherapy with least side effects.

## Figures and Tables

**Table 1:** miRNAs involved in immunity and immunotherapy for gastroenterological cancers.

Cancer types/cell type	MicroRNAs	Target genes	Immunological component/process	References
**Hepatocellular Carcinoma**	miR-34a	DAPK2/SP1 pathway	Dendritic cell activation	[74]
	miR-34a	PD-L1	PD-1/PD-L1 immune checkpoint blockade	[75]
	miR-146a	Effector of STAT3	NK cells and anti-tumor lymphocytes	[76]
	miR-20a, miR-93 and miR-106b	MICA/B	Antigen presentation and immune evasion	[79]
	miR-101	DUSP1	Proinflammatory factors and tumor-associated macrophages	[82]
	miR-26a	Macrophage colony-stimulating factor (M-CSF)	Chemokine ligand (CCL) 22, IL10 and macrophage infiltration	[84]
	miR-122	HCV genome	Assisting replication of HCV	[86]
	miR-122	Cyclin G_1_	Inhibiting transcription of HBV	[88]
	miR-122	Suppressor of cytokine signaling 3 (SOCS3)	Interferon production	[89]
	miR-122	Chemokine (C-C motif) ligand 2 (CCL2)	CCR2^+^CD11b^high^Gr1^+^ immune cells recruitment, proinflammatory cytokine production	[90]
	miR-185	LDLR, SCD1, SCARB1 and SREBP2	HCV replication	[91]
	miR-130b	LDLR, lipid metabolism pathway	HCV replication; reinforcing the antiviral activity of 25-hydroxycholesterol (25-HC),	[91]

**Pancreatic Cancer**	miR-203	Toll-Like Receptor 4	Modulating TLR-mediated immune response and facilitate immune escape	[92]
	miR-142-5p	PD-L1	PD-1/PD-L1 immune checkpoint blockade	[93]
	miR-454	Stromal cell derived factor-1	Macrophage recruitment and dendritic cell maturation	[94]
	miR-206	Chemokines (C-X-C motif) ligand 1 and (C-C motif) ligand 2, Interleukin-8 and the granulocyte macrophage colony-stimulating factor	Inhibiting inflammation and immune reaction	[95]
	miR-206	Vascular endothelial growth factor C	Inhibiting blood and lymphatic vessel formation	[95]
	miR-9	unknown	Inflammation of acute pancreatitis	[96]
	miR-216a	PTEN, Smad7, pAkt and TGF-*β* receptor 1	Inflammation of acute pancreatitis	[97]
	miR-146a	IL-1b, IL-6 and TNF-a	Inflammation of chronic pancreatitis	[98]

**Colorectal Cancer**	miR-222 and -339	ICAM-1	Promotes cytotoxicity of CTL's	[99]
	miR-21	PDCD4	IL-10 transcription and CD3^+^ and CD45RO^+^ cell selection	[100]
	miR-17-5p and -20a	STAT3	Decreased burden from reactive oxygen species and inhibition of MDSC immunosuppression	[101, 102]
	miR-124	STAT3	Decreased progression of ulcerative colitis	[103]
	miR-19b	HIF-alpha	Anti-inflammatory in Crohn's disease	[104]
	miR-494	PTEN	Increased number of pro-cancer MDSC's	
	miR-484 and -19a	CD137L	Decreased PI3K/mTOR signaling and IL-8 production	[105]
	miR-142-5p	PD-L1	Increased viability of CTL's	[93]
	miR-20b, -21, and -130b	PTEN	PD-L1 overexpression and CRC proliferation	[101, 106]

**Gastric Cancer**	miR-34a	DAPK2/SP1	Increased dendritic cell immune response	[74]

**Gallbladder Cancer**	miR-218-5p	Downregulated by lncRNA CCAT1	Modulation of the tumor micro-environment	[107]
	miR-155	IFN*γ*	Activation and proliferation of CD4^+^ and CD8^+^ T cells	[108]
	miR-133a-3p	Recombination signal-binding protein J*κ*	Differentiation of T cell lineage from common lymphoid precursor.	[109]
	miR-1, miR-133, miR-143 and miR-145	VEGF-A, ErbB3, AXL	Modulation of the tumor micro-environment	[110]

**Esophageal Cancer**	miR-31	STK40	Inflammatory signaling	[111]
	miR-34a	Upregulated by NF-kappaB	Inflammation signaling	[112]
	miR-155	SOCS1/JAK/STAT pathway	Normal function of Kupffer cells	[113]
	miR-21	Exosomal miRNA	T cells and macrophages activation	[114]

## References

[1] Siegel R. L., Miller K. D., Jemal A. (2017). Cancer statistics, 2017. *CA: A Cancer Journal for Clinicians*.

[2] Hurwitz H. (2004). Integrating the anti-VEGF - A humanized monoclonal antibody bevacizumab with chemotherapy in advanced colorectal cancer. *Clinical Colorectal Cancer*.

[3] Dutton S. J., Ferry D. R., Blazeby J. M. (2014). Gefitinib for oesophageal cancer progressing after chemotherapy (COG): a phase 3, multicentre, double-blind, placebo-controlled randomised trial. *The Lancet Oncology*.

[4] Bang Y.-J., Van Cutsem E., Feyereislova A. (2010). Trastuzumab in combination with chemotherapy versus chemotherapy alone for treatment of HER2- positive advanced gastric or gastro-oesophageal junction cancer (ToGA): a phase 3, open-label, randomised controlled trial. *The Lancet*.

[5] Lubner S. J., Uboha N. V., Deming D. A. (2017). Primary and acquired resistance to biologic therapies in gastrointestinal cancers. *Journal of Gastrointestinal Oncology*.

[6] Burris H. A., Moore M. J., Andersen J. (1997). Improvements in survival and clinical benefit with gemcitabine as first- line therapy for patients with advanced pancreas cancer: a randomized trial. *Journal of Clinical Oncology*.

[7] Amrutkar M., Gladhaug I. P. (2017). Pancreatic cancer chemoresistance to gemcitabine. *Cancers*.

[8] Llovet J. M., Ricci S., Mazzaferro V. (2008). Sorafenib in Advanced Hepatocellular Carcinoma. *The New England Journal of Medicine*.

[9] van der Bruggen P., Traversari C., Chomez P. (1991). A gene encoding an antigen recognized by cytolytic T lymphocytes on a human melanoma. *Science*.

[10] Kumai T. (2017). Peptide vaccines in cancer-old concept revisited. *Current Opinion in Immunology*.

[11] Yang J. C., Rosenberg S. A. (2016). Adoptive T-Cell Therapy for Cancer. *Advances in Immunology*.

[12] Takeuchi Y., Nishikawa H. (2016). Roles of regulatory T cells in cancer immunity. *International Immunology*.

[13] Park S. J., Nakagawa T., Kitamura H. (2004). IL-6 regulates in vivo dendritic cell differentiation through STAT3 activation. *The Journal of Immunology*.

[14] Kitamura H., Ohno Y., Toyoshima Y. (2017). Interleukin-6/STAT3 signaling as a promising target to improve the efficacy of cancer immunotherapy. *Cancer Science*.

[15] Tanaka A., Sakaguchi S. (2017). Regulatory T cells in cancer immunotherapy. *Cell Research*.

[16] Bronte V., Brandau S., Chen S.-H. (2016). Recommendations for myeloid-derived suppressor cell nomenclature and characterization standards. *Nature Communications*.

[17] Sumida K., Wakita D., Narita Y. (2012). Anti-IL-6 receptor mAb eliminates myeloid-derived suppressor cells and inhibits tumor growth by enhancing T-cell responses. *European Journal of Immunology*.

[18] Agata Y., Kawasaki A., Nishimura H. (1996). Expression of the PD-1 antigen on the surface of stimulated mouse T and B lymphocytes. *International Immunology*.

[19] Freeman G. J., Long A. J., Iwai Y. (2000). Engagement of the PD-1 immunoinhibitory receptor by a novel B7 family member leads to negative regulation of lymphocyte activation. *The Journal of Experimental Medicine*.

[20] Latchman Y., Wood C. R., Chernova T. (2001). PD-L2 is a second ligand for PD-1 and inhibits T cell activation. *Nature Immunology*.

[21] Okazaki T., Maeda A., Nishimura H., Kurosaki T., Honjo T. (2001). PD-1 immunoreceptor inhibits B cell receptor-mediated signaling by recruiting src homology 2-domain-containing tyrosine phosphatase 2 to phosphotyrosine. *Proceedings of the National Acadamy of Sciences of the United States of America*.

[22] Parry R. V., Chemnitz J. M., Frauwirth K. A. (2005). CTLA-4 and PD-1 receptors inhibit T-cell activation by distinct mechanisms. *Molecular and Cellular Biology*.

[23] Nishimura H., Honjo T., Minato N. (2000). Facilitation of *β* selection and modification of positive selection in the thymus of PD-1-deficient mice. *The Journal of Experimental Medicine*.

[24] Probst H. C., McCoy K., Okazaki T., Honjo T., van den Broek M. (2005). Resting dendritic cells induce peripheral CD8^+^ T cell tolerance through PD-1 and CTLA-4. *Nature Immunology*.

[25] Ahmadzadeh M. (2009). OR.93. Tumor Antigen-specific CD8 T Cells Infiltrating the Tumor Express High Levels of PD-1 and are Functionally Impaired. *Clinical Immunology*.

[26] Lee S., Seo S., Kim B. (2006). Corrigendum to “IFN-gamma regulates the expression of B7-H1 in dermal fibroblast cells” [J. Dermatol. Sci. 40 (2005) 95–103]. *Journal of Dermatological Science*.

[27] Wu C., Zhu Y., Jiang J., Zhao J., Zhang X.-G., Xu N. (2006). Immunohistochemical localization of programmed death-1 ligand-1 (PD-L1) in gastric carcinoma and its clinical significance. *Acta Histochemica*.

[28] Hou J., Yu Z., Xiang R. (2014). Correlation between infiltration of FOXP3+ regulatory T cells and expression of B7-H1 in the tumor tissues of gastric cancer. *Experimental and Molecular Pathology*.

[29] Le D. T., Uram J. N., Wang H. (2015). PD-1 Blockade in Tumors with Mismatch-Repair Deficiency. *The New England Journal of Medicine*.

[30] Sabbatino F., Villani V., Yearley J. H. (2016). PD-L1 and HLA class i antigen expression and clinical course of the disease in intrahepatic cholangiocarcinoma. *Clinical Cancer Research*.

[31] Walter D., Herrmann E., Schnitzbauer A. A. (2017). PD-L1 expression in extrahepatic cholangiocarcinoma. *Histopathology*.

[32] Calderaro J., Rousseau B., Amaddeo G. (2016). Programmed death ligand 1 expression in hepatocellular carcinoma: Relationship With clinical and pathological features. *Hepatology*.

[33] Wang L., Ma Q., Chen X., Guo K., Li J., Zhang M. (2010). Clinical significance of B7-H1 and B7-1 expressions in pancreatic carcinoma. *World Journal of Surgery*.

[34] Zheng P., Zhou Z. (2015). Human Cancer Immunotherapy with PD-1/PD-L1 Blockade. *Biomarkers in Cancer*.

[35] Finkelmeier F., Canli Ö., Tal A. (2016). High levels of the soluble programmed death-ligand (sPD-L1) identify hepatocellular carcinoma patients with a poor prognosis. *European Journal of Cancer*.

[36] Thompson R. H. (2004). Costimulatory B7-H1 in renal cell carcinoma patients: Indicator of tumor aggressiveness and potential therapeutic target. *Proceedings of the National Academy of Sciences of the United States*.

[37] Hamanishi J. (2007). Programmed cell death 1 ligand 1 and tumor-infiltrating CD8+ T lymphocytes are prognostic factors of human ovarian cancer. *Proceedings of the National Academy of Sciences of the United States*.

[38] Iwai Y., Ishida M., Tanaka Y., Okazaki T., Honjo T., Minato N. (2002). Involvement of PD-L1 on tumor cells in the escape from host immune system and tumor immunotherapy by PD-L1 blockade. *Proceedings of the National Acadamy of Sciences of the United States of America*.

[39] Brahmer J. R., Drake C. G., Wollner I. (2010). Phase I study of single-agent anti-programmed death-1 (MDX-1106) in refractory solid tumors: safety, clinical activity, pharmacodynamics, and immunologic correlates. *Journal of Clinical Oncology*.

[40] Muro K., Chung H. C., Shankaran V. (2016). Pembrolizumab for patients with PD-L1-positive advanced gastric cancer (KEYNOTE-012): a multicentre, open-label, phase 1b trial. *The Lancet Oncology*.

[41] Buchbinder E. I., Desai A. (2015). CTLA-4 and PD-1 pathways: similarities, differences, and implications of their inhibition. *American Journal of Clinical Oncology*.

[42] Wolchok J. D., Saenger Y. (2008). The mechanism of anti-CTLA-4 activity and the negative regulation of T-cell activation.. *The Oncologist*.

[43] Chang C.-C., Campoli M., Ferrone S. (2004). HLA class I antigen expression in malignant cells: Why does it not always correlate with CTL-mediated lysis?. *Current Opinion in Immunology*.

[44] Nobuoka D., Yoshikawa T., Fujiwara T., Nakatsura T. (2013). Peptide intra-tumor injection for cancer immunotherapy: Enhancement of tumor cell antigenicity is a novel and attractive strategy. *Human Vaccines & Immunotherapeutics*.

[45] Farazi T. A., Juranek S. A., Tuschl T. (2008). The growing catalog of small RNAs and their association with distinct Argonaute/Piwi family members. *Development*.

[46] Friedman R. C., Farh K. K., Burge C. B., Bartel D. P. (2009). Most mammalian mRNAs are conserved targets of microRNAs. *Genome Research*.

[47] Wienholds E., Kloosterman W. P., Miska E. (2005). MicroRNA expression in zebrafish embryonic development. *Science*.

[48] Wan G., Mathur R., Hu X., Zhang X., Lu X. (2011). miRNA response to DNA damage. *Trends in Biochemical Sciences*.

[49] Liu G., Sun Y., Ji P. (2014). MiR-506 suppresses proliferation and induces senescence by directly targeting the CDK4/6-FOXM1 axis in ovarian cancer. *The Journal of Pathology*.

[50] Chen C., Li L., Lodish H. F., Bartel D. P. (2004). MicroRNAs modulate hematopoietic lineage differentiation. *Science*.

[51] Baehrecke E. H. (2003). miRNAs: Micro managers of programmed cell death. *Current Biology*.

[52] Hommers L. G., Domschke K., Deckert J. (2015). Heterogeneity and Individuality: microRNAs in Mental Disorders. *Journal of Neural Transmission*.

[53] Lewohl J. M., Nunez Y. O., Dodd P. R., Tiwari G. R., Harris R. A., Mayfield R. D. (2011). Up-regulation of microRNAs in brain of human alcoholics. *Alcoholism: Clinical and Experimental Research*.

[54] Frost R. J. A., Olson E. N. (2011). Control of glucose homeostasis and insulin sensitivity by the Let-7 family of microRNAs. *Proceedings of the National Acadamy of Sciences of the United States of America*.

[55] Garzon R., Fabbri M., Cimmino A., Calin G. A., Croce C. M. (2006). MicroRNA expression and function in cancer. *Trends in Molecular Medicine*.

[56] Zhou R., O'Hara S. P., Chen X.-M. (2011). MicroRNA regulation of innate immune responses in epithelial cells. *Cellular & Molecular Immunology*.

[57] Kim T.-D., Lee S. U., Yun S. (2011). Human microRNA-27a∗targets Prf1 and GzmB expression to regulate NK-cell cytotoxicity. *Blood*.

[58] Gong J., Liu R., Zhuang R. (2012). MiR-30c-1∗ promotes natural killer cell cytotoxicity against human hepatoma cells by targeting the transcription factor HMBOX1. *Cancer Science*.

[59] Squadrito M. L., Pucci F., Magri L. (2012). MiR-511-3p modulates genetic programs of tumor-associated macrophages. *Cell Reports*.

[60] Tili E., Michaille J. J., Cimino A. (2007). Modulation of miR-155 and miR-125b levels following lipopolysaccharide/TNF-*α* stimulation and their possible roles in regulating the response to endotoxin shock. *The Journal of Immunology*.

[61] Hwang S.-Y., Sun H.-Y., Lee K.-H. (2012). 5′-Triphosphate-RNA-independent activation of RIG-I via RNA aptamer with enhanced antiviral activity. *Nucleic Acids Research*.

[62] Hoefig K. P., Heissmeyer V. (2008). MicroRNAs grow up in the immune system. *Current Opinion in Immunology*.

[63] Lin Y.-C., Kuo M.-W., Yu J. (2008). c-Myb is an evolutionary conserved miR-150 target and miR-150/c-Myb interaction is important for embryonic development. *Molecular Biology and Evolution*.

[64] Rao D. S., O'Connell R. M., Chaudhuri A. A., Garcia-Flores Y., Geiger T. L., Baltimore D. (2010). MicroRNA-34a perturbs B lymphocyte development by repressing the forkhead box transcription factor Foxp1. *Immunity*.

[65] Costinean S., Sandhu S. K., Pedersen I. M. (2009). Src homology 2 domain-containing inositol-5-phosphatase and CCAAT enhancer-binding protein *β* are targeted by miR-155 in B cells of E*μ*-*MiR*-155 transgenic mice. *Blood*.

[66] Matsuyama H., Suzuki H. I., Nishimori H. (2011). miR-135b mediates NPM-ALK-driven oncogenicity and renders IL-17-producing immunophenotype to anaplastic large cell lymphoma. *Blood*.

[67] Himmelreich H., Mathys A., Wodnar-Filipowicz A., Kalberer C. P. (2011). Post-transcriptional regulation of ULBP1 ligand for the activating immunoreceptor NKG2D involves 3' untranslated region. *Human Immunology*.

[68] Zhang Y., Sun E., Li X. (2017). miR-155 contributes to Df1-induced asthma by increasing the proliferative response of Th cells via CTLA-4 downregulation. *Cellular Immunology*.

[69] El-Serag H. B., Rudolph K. L. (2007). Hepatocellular carcinoma: epidemiology and molecular carcinogenesis. *Gastroenterology*.

[70] Rahib L., Smith B. D., Aizenberg R., Rosenzweig A. B., Fleshman J. M., Matrisian L. M. (2014). Projecting cancer incidence and deaths to 2030: the unexpected burden of thyroid, liver, and pancreas cancers in the United States. *Cancer Research*.

[71] Bruix J., Takayama T., Mazzaferro V. (2015). Adjuvant sorafenib for hepatocellular carcinoma after resection or ablation (STORM): A phase 3, randomised, double-blind, placebo-controlled trial. *The Lancet Oncology*.

[72] Wang X., Li J., Dong K. (2015). Tumor suppressor miR-34a targets PD-L1 and functions as a potential immunotherapeutic target in acute myeloid leukemia. *Cellular Signalling*.

[73] Xu S., Tao Z., Hai B. (2016). miR-424(322) reverses chemoresistance via T-cell immune response activation by blocking the PD-L1 immune checkpoint. *Nature Communications*.

[74] Yan L.-H., Chen Z.-N., Li L. (2016). E2F-1 promotes DAPK2-induced anti-tumor immunity of gastric cancer cells by targeting miR-34a. *Tumor Biology*.

[75] Cortez M. A. (2016). PDL1 Regulation by p53 via miR-34. *Journal of the National Cancer Institute*.

[76] Sun X., Sui Q., Zhang C., Tian Z., Zhang J. (2013). Targeting blockage of stat3 in hepatocellular carcinoma cells augments nk cell functions via reverse hepatocellular carcinoma-induced immune suppression. *Molecular Cancer Therapeutics*.

[77] Wang T., Niu G., Kortylewski M. (2004). Regulation of the innate and adaptive immune responses by Stat-3 signaling in tumor cells. *Nature Medicine*.

[78] Yu H., Pardoll D., Jove R. (2009). STATs in cancer inflammation and immunity: a leading role for STAT3. *Nature Reviews Cancer*.

[79] Stern-Ginossar N., Gur C., Biton M. (2008). Human microRNAs regulate stress-induced immune responses mediated by the receptor NKG2D. *Nature Immunology*.

[80] Armeanu S., Bitzer M., Lauer U. M. (2005). Natural killer cell-mediated lysis of hepatoma cells via specific induction of NKG2D ligands by the histone deacetylase inhibitor sodium valproate. *Cancer Research*.

[81] Yang H., Lan P., Hou Z. (2015). Histone deacetylase inhibitor SAHA epigenetically regulates miR-17-92 cluster and MCM7 to upregulate MICA expression in hepatoma. *British Journal of Cancer*.

[82] Wei X., Tang C., Lu X. (2015). MiR-101 targets DUSP1 to regulate the TGF-ß secretion in sorafenib inhibits macrophage-induced growth of hepatocarcinoma. *Oncotarget *.

[83] Qian H., Yang C., Yang Y. (2017). MicroRNA-26a inhibits the growth and invasiveness of malignant melanoma and directly targets on MITF gene. *Cell Death Discovery*.

[84] Chai Z.-T., Zhu X.-D., Ao J.-Y. (2015). MicroRNA-26a suppresses recruitment of macrophages by down-regulating macrophage colony-stimulating factor expression through the PI3K/Akt pathway in hepatocellular carcinoma. *Journal of Hematology & Oncology*.

[85] El-Serag H. B. (2012). Epidemiology of viral hepatitis and hepatocellular carcinoma. *Gastroenterology*.

[86] Jopling C. L., Yi M., Lancaster A. M., Lemon S. M., Sarnow P. (2005). Molecular biology: modulation of hepatitis C virus RNA abundance by a liver-specific MicroRNA. *Science*.

[87] Janssen H. L. A., Reesink H. W., Lawitz E. J. (2013). Treatment of HCV infection by targeting microRNA. *The New England Journal of Medicine*.

[88] Wang S., Qiu L., Yan X. (2012). Loss of microRNA 122 expression in patients with hepatitis B enhances hepatitis B virus replication through cyclin G 1-modulated P53 activity. *Hepatology*.

[89] Yoshikawa T., Takata A., Otsuka M. (2012). Silencing of microRNA-122 enhances interferon-*α* signaling in the liver through regulating SOCS3 promoter methylation. *Scientific Reports*.

[90] Li C., Deng M., Hu J. (2016). Chronic inflammation contributes to the development of hepatocellular carcinoma by decreasing miR-122 levels. *Oncotarget *.

[91] Singaravelu R., O'Hara S., Jones D. M. (2015). MicroRNAs regulate the immunometabolic response to viral infection in the liver. *Nature Chemical Biology*.

[92] Zhou M., Chen J., Zhou L., Chen W., Ding G., Cao L. (2014). Pancreatic cancer derived exosomes regulate the expression of TLR4 in dendritic cells via miR-203. *Cellular Immunology*.

[93] Jia L., Xi Q., Wang H. (2017). miR-142-5p regulates tumor cell PD-L1 expression and enhances anti-tumor immunity. *Biochemical and Biophysical Research Communications*.

[94] Fan Y., Shi C., Li T., Kuang T. (2017). microRNA-454 shows anti-angiogenic and anti-metastatic activity in pancreatic ductal adenocarcinoma by targeting LRP6. *American Journal of Cancer Research*.

[95] Keklikoglou I., Hosaka K., Bender C. (2015). MicroRNA-206 functions as a pleiotropic modulator of cell proliferation, invasion and lymphangiogenesis in pancreatic adenocarcinoma by targeting ANXA2 and KRAS genes. *Oncogene*.

[96] Qian D., Wei G., Xu C. (2017). Bone marrow-derived mesenchymal stem cells (BMSCs) repair acute necrotized pancreatitis by secreting microRNA-9 to target the NF-*κ*B1/p50 gene in rats. *Scientific Reports*.

[97] Zhang J., Ning X., Cui W., Bi M., Zhang D., Zhang J. (2015). Transforming Growth Factor (TGF)-*β*-Induced MicroRNA-216a Promotes Acute Pancreatitis Via Akt and TGF-*β* Pathway in Mice. *Digestive Diseases and Sciences*.

[98] El Gazzar M., Church A., Liu T. F., McCall C. E. (2011). MicroRNA-146a regulates both transcription silencing and translation disruption of TNF-*α* during TLR4-induced gene reprogramming. *Journal of Leukocyte Biology*.

[99] Ueda R., Kohanbash G., Sasaki K. (2009). Dicer-regulated microRNAs 222 and 339 promote resistance of cancer cells to cytotoxic T-lymphocytes by down-regulation of ICAM-1. *Proceedings of the National Acadamy of Sciences of the United States of America*.

[100] Mima K., Nishihara R., Qian Z., Baba H., Ogino S. (2016). 173PD MicroRNA MIR21, T cells, and PTGS2 expression in Colorectal Cancer. *Annals of Oncology*.

[101] Li X., Nie J., Mei Q., Han W.-D. (2016). MicroRNAs: novel immunotherapeutic targets in colorectal carcinoma. *World Journal of Gastroenterology*.

[102] Zhang M., Liu Q., Mi S. (2011). Both miR-17-5p and miR-20a alleviate suppressive potential of myeloid-derived suppressor cells by modulating STAT3 expression. *The Journal of Immunology*.

[103] Koukos G., Polytarchou C., Kaplan J. L. (2013). MicroRNA-124 regulates STAT3 expression and is down-regulated in colon tissues of pediatric patients with ulcerative colitis. *Gastroenterology*.

[104] Wang H., Flach H., Onizawa M., Wei L., Mcmanus M. T., Weiss A. (2014). Negative regulation of Hif1a expression and TH17 differentiation by the hypoxia-regulated microRNA miR-210. *Nature Immunology*.

[105] Yu H. W. H., Sze D. M. Y., Cho W. C. S. (2013). MicroRNAs involved in anti-tumour immunity. *International Journal of Molecular Sciences*.

[106] Zhu J., Chen L., Zou L. (2014). MiR-20b, -21, and -130b inhibit PTEN expression resulting in B7-H1 over-expression in advanced colorectal cancer. *Human Immunology*.

[107] Ma M.-Z., Chu B.-F., Zhang Y. (2015). Long non-coding RNA CCAT1 promotes gallbladder cancer development via negative modulation of miRNA-218-5p. *Cell Death & Disease*.

[108] Huffaker T. B., Lee S.-H., Tang W. W. (2017). Antitumor immunity is defective in T cell–specific microRNA-155– deficient mice and is rescued by immune checkpoint blockade. *The Journal of Biological Chemistry*.

[109] Huang Y., Wu Y., Dong J., Han D., Yang S., Jiang L. (2016). MicroRNA-133a-3p exerts inhibitory effects on gallbladder carcinoma via targeting RBPJ. *American Journal of Cancer Research*.

[110] Letelier P. (1849). miR-1 and miR-145 act as tumor suppressor microRNAs in gallbladder cancer. *International Journal of Clinical and Experimental Pathology*.

[111] Taccioli C., Garofalo M., Chen H. (2015). Repression of esophageal neoplasia and inflammatory signaling by anti-MIR-31 delivery in vivo. *Journal of the National Cancer Institute*.

[112] Li J., Wang K., Chen X. (2012). Transcriptional activation of microRNA-34a by NF-kappaB in human esophageal cancer cells. *BMC Molecular Biology*.

[113] Li J., Gong J., Li P. (2014). Knockdown of MicroRNA-155 in kupffer cells results in immunosuppressive effects and prolongs survival of mouse liver allografts. *Transplantation*.

[114] Tanaka Y., Kamohara H., Kinoshita K. (2013). Clinical impact of serum exosomal microRNA-21 as a clinical biomarker in human esophageal squamous cell carcinoma. *Cancer*.

[115] Chang J., Nicolas E., Marks D. (2004). miR-122, a mammalian liver-specific microRNA, is processed from hcr mRNA and may downregulate the high affinity cationic amino acid transporter CAT-1. *RNA Biology*.

[116] Landgraf P. (2007). *A mammalian microRNA expression atlas based on small RNA library sequencing*.

[117] Jopling C. L. (2012). Liver-specific microRNA-122: biogenesis and function. *RNA Biology*.

[118] Szabo G., Bala S. (2013). MicroRNAs in liver disease. *Nature Reviews Gastroenterology & Hepatology*.

[119] Kutay H., Bai S., Datta J. (2006). Downregulation of miR-122 in the rodent and human hepatocellular carcinomas. *Journal of Cellular Biochemistry*.

[120] Tsai W.-C., Hsu S.-D., Hsu C.-S. (2012). MicroRNA-122 plays a critical role in liver homeostasis and hepatocarcinogenesis. *The Journal of Clinical Investigation*.

[121] Coulouarn C., Factor V. M., Andersen J. B., Durkin M. E., Thorgeirsson S. S. (2009). Loss of miR-122 expression in liver cancer correlates with suppression of the hepatic phenotype and gain of metastatic properties. *Oncogene*.

[122] Hsu S.-H., Wang B., Kota J. (2012). Essential metabolic, anti-inflammatory, and anti-tumorigenic functions of miR-122 in liver. *The Journal of Clinical Investigation*.

[123] Padgett K. A., Lan R. Y., Leung P. C. (2009). Primary biliary cirrhosis is associated with altered hepatic microRNA expression. *Journal of Autoimmunity*.

[124] Jemal A., Ward E. M., Johnson C. J. (2017). Annual Report to the Nation on the Status of Cancer, 1975-2014, Featuring Survival. *Journal of the National Cancer Institute*.

[125] Admyre C., Johansson S. M., Paulie S., Gabrielsson S. (2006). Direct exosome stimulation of peripheral human T cells detected by ELISPOT. *European Journal of Immunology*.

[126] Raposo G., Nijman H. W., Stoorvogel W. (1996). B lymphocytes secrete antigen-presenting vesicles. *The Journal of Experimental Medicine*.

[127] Théry C., Regnault A., Garin J. (1999). Molecular characterization of dendritic cell-derived exosomes. Selective accumulation of the heat shock protein hsc73. *The Journal of Cell Biology*.

[128] Wolfers J., Lozier A., Raposo G. (2001). Tumor-derived exosomes are a source of shared tumor rejection antigens for CTL cross-priming. *Nature Medicine*.

[129] Tan A., de la Peña H., Seifalian A. M. (2010). The application of exosomes as a nanoscale cancer vaccine. *International Journal of Nanomedicine*.

[130] Valadi H., Ekström K., Bossios A., Sjöstrand M., Lee J. J., Lötvall J. O. (2007). Exosome-mediated transfer of mRNAs and microRNAs is a novel mechanism of genetic exchange between cells. *Nature Cell Biology*.

[131] Théry C., Ostrowski M., Segura E. (2009). Membrane vesicles as conveyors of immune responses. *Nature Reviews Immunology*.

[132] Que R.-S., Lin C., Ding G.-P., Wu Z.-R., Cao L.-P. (2016). Increasing the immune activity of exosomes: the effect of miRNA-depleted exosome proteins on activating dendritic cell/cytokine-induced killer cells against pancreatic cancer. *Journal of Zhejiang University SCIENCE B*.

[133] Du J., Wang J., Tan G. (2012). Aberrant elevated microRNA-146a in dendritic cells (DC) induced by human pancreatic cancer cell line BxPC-3-conditioned medium inhibits DC maturation and activation. *Medical Oncology*.

[134] Meng S., Wang H., Xue D., Zhang W. (2017). Screening and validation of differentially expressed extracellular miRNAs in acute pancreatitis. *Molecular Medicine Reports*.

[135] Zhang X.-X., Deng L.-H., Chen W.-W. (2017). Circulating microRNA 216 as a Marker for the Early Identification of Severe Acute Pancreatitis. *The American Journal of the Medical Sciences*.

[136] Whitcomb D. C., Frulloni L., Garg P. (2016). Chronic pancreatitis: An international draft consensus proposal for a new mechanistic definition. *Pancreatology*.

[137] Kleeff J., Whitcomb D. C., Shimosegawa T. (2017). Chronic pancreatitis. *Nature Reviews Disease Primers*.

[138] Bang U. C., Benfield T., Hyldstrup L., Bendtsen F., Beck Jensen J.-E. (2014). Mortality, cancer, and comorbidities associated with chronic pancreatitis: A Danish nationwide matched-cohort study. *Gastroenterology*.

[139] Wang Z., Li D., Jin J., Wang Q., Zhao S., Bai Y. (2016). Association between microRNA polymorphisms and chronic pancreatitis. *Pancreatology*.

[140] Cobb B. S., Hertweck A., Smith J. (2006). A role for Dicer in immune regulation. *The Journal of Experimental Medicine*.

[141] de Vries J. E., Yssel H., Spits H. (1989). Interplay between the TCR/CD3 Complex and CD4 or CD8 in the Activation of Cytotoxic T Lymphocytes. *Immunological Reviews*.

[142] Hotta K., Sho M., Fujimoto K. (2011). Prognostic significance of CD45RO memory T cells in renal cell carcinoma. *British Journal of Cancer*.

[143] Srivastava M. K., Sinha P., Clements V. K., Rodriguez P., Ostrand-Rosenberg S. (2010). Myeloid-derived suppressor cells inhibit T-cell activation by depleting cystine and cysteine. *Cancer Research*.

[144] Liu Y., Lai L., Chen Q. (2012). MicroRNA-494 is required for the accumulation and functions of tumor-expanded myeloid-derived suppressor cells via targeting of PTEN. *The Journal of Immunology*.

[145] Grivennikov S. I. (2013). Inflammation and colorectal cancer: colitis-associated neoplasia. *Seminars in Immunopathology*.

[146] Cheng X., Zhang X., Su J. (2015). MiR-19b downregulates intestinal SOCS3 to reduce intestinal inflammation in Crohn's disease. *Scientific Reports*.

[147] Heinsbroek S. E. M., Squadrito M. L., Schilderink R. (2016). MiR-511-3p, embedded in the macrophage mannose receptor gene, contributes to intestinal inflammation. *Mucosal Immunology*.

[148] Coffelt S. B., Wellenstein M. D., De Visser K. E. (2016). Neutrophils in cancer: Neutral no more. *Nature Reviews Cancer*.

[149] Gabrilovich D. I., Ostrand-Rosenberg S., Bronte V. (2012). Coordinated regulation of myeloid cells by tumours. *Nature Reviews Immunology*.

[150] Tazzyman S., Niaz H., Murdoch C. (2013). Neutrophil-mediated tumour angiogenesis: Subversion of immune responses to promote tumour growth. *Seminars in Cancer Biology*.

[151] Noy R., Pollard J. W. (2014). Tumor-associated macrophages: from mechanisms to therapy. *Immunity*.

[152] Mei Q., Xue G., Li X. (2015). Methylation-induced loss of miR-484 in microsatellite-unstable colorectal cancer promotes both viability and IL-8 production via CD137L. *The Journal of Pathology*.

[153] Chen B. (2013). Role of miR-19a targeting TNF-alpha in mediating ulcerative colitis. *Scandinavian Journal of Gastroenterology*.

[154] Patel S. P., Kurzrock R. (2015). PD-L1 expression as a predictive biomarker in cancer immunotherapy. *Molecular Cancer Therapeutics*.

[155] Siegel R., Ma J., Zou Z., Jemal A. (2014). Cancer statistics, 2014. *CA: A Cancer Journal for Clinicians*.

[156] Anderson W. F., Camargo M. C., Fraumeni J. F., Correa P., Rosenberg P. S., Rabkin C. S. (2010). Age-specific trends in incidence of noncardia gastric cancer in US adults. *Journal of the American Medical Association*.

[157] Ginsberg D. (2002). E2F1 pathways to apoptosis. *FEBS Letters*.

[158] Li Z., Yu X., Shen J., Law P. T. Y., Chan M. T. V., Wu W. K. K. (2015). MicroRNA expression and its implications for diagnosis and therapy of gallbladder cancer. *Oncotarget *.

[159] Chapman B. C., Jones T., McManus M. C., Shah R., Gajdos C. (2014). Metastatic papillary gallbladder carcinoma with a unique presentation and clinical course. *JOP*.

[160] Kono H., Nakamura M., Ohtsuka T. (2013). High expression of microRNA-155 is associated with the aggressive malignant behavior of gallbladder carcinoma. *Oncology Reports*.

[161] Li Y., Zhang J., Ma H. (2014). Chronic inflammation and gallbladder cancer. *Cancer Letters*.

[162] Savard C. E., Blinman T. A., Choi H., Lee S., Pandol S. J., Lee S. P. (2002). Expression of cytokine and chemokine mRNA and secretion of tumor necrosis factor-*α* by gallbladder epithelial cells: Response to bacterial lipopolysaccharides. *BMC Gastroenterology*.

[163] Xiong L., Yang Z., Yang L., Liu J., Miao X. (2012). Expressive levels of MUC1 and MUC5AC and their clinicopathologic significances in the benign and malignant lesions of gallbladder. *Journal of Surgical Oncology*.

[164] Tsuchida N., Ryder T., Ohtsubo E. (1982). Nucleotide sequence of the oncogene encoding the p21 transforming protein of Kirsten murine sarcoma virus. *Science*.

[165] Sundaram G. M., Bramhachari P. V. (2017). Molecular interplay of pro-inflammatory transcription factors and non-coding RNAs in esophageal squamous cell carcinoma. *Tumor Biology*.

[166] Wu W., Bhagat T. D., Yang X. (2013). Hypomethylation of noncoding DNA regions and overexpression of the long noncoding RNA, AFAP1-AS1, in Barrett's esophagus and esophageal adenocarcinoma. *Gastroenterology*.

[167] Wang X., Li M., Wang Z. (2015). Silencing of long noncoding rna malat1 by mir-101 and mir-217 inhibits proliferation, migration, and invasion of esophageal squamous cell carcinoma cells. *The Journal of Biological Chemistry*.

[168] Liu S. G., Qin X. G., Zhao B. S. (2013). Differential expression of miRNAs in esophageal cancer tissue. *Oncology Letters*.

[169] Koumangoye R. B., Andl T., Taubenslag K. J. (2015). SOX4 interacts with EZH2 and HDAC3 to suppress microRNA-31 in invasive esophageal cancer cells. *Molecular Cancer*.

[170] Akanuma N., Hoshino I., Akutsu Y. (2014). MicroRNA-133a regulates the mRNAs of two invadopodia-related proteins, FSCN1 and MMP14, in esophageal cancer. *British Journal of Cancer*.

[171] Chen Z.-L., Zhao X.-H., Wang J.-W. (2011). microRNA-92a promotes lymph node metastasis of human esophageal squamous cell carcinoma via E-cadherin. *The Journal of Biological Chemistry*.

[172] Tian Y., Luo A., Cai Y. (2010). MicroRNA-10b promotes migration and invasion through KLF4 in human esophageal cancer cell lines. *The Journal of Biological Chemistry*.

[173] Ye Z., Fang J., Dai S. (2016). MicroRNA-34a induces a senescence-like change via the down-regulation of SIRT1 and up-regulation of p53 protein in human esophageal squamous cancer cells with a wild-type p53 gene background. *Cancer Letters*.

[174] Hiyoshi Y., Kamohara H., Karashima R. (2009). MicroRNA-21 regulates the proliferation and invasion in esophageal squamous cell carcinoma. *Clinical Cancer Research*.

[175] Kono K., Kawaida H., Takahashi A. (2006). CD4(+)CD25^high^ regulatory T cells increase with tumor stage in patients with gastric and esophageal cancers. *Cancer Immunology, Immunotherapy*.

[176] Curiel T. J., Coukos G., Zou L. (2004). Specific recruitment of regulatory T cells in ovarian carcinoma fosters immune privilege and predicts reduced survival. *Nature Medicine*.

[177] Gabitass R. F., Annels N. E., Stocken D. D., Pandha H. A., Middleton G. W. (2011). Elevated myeloid-derived suppressor cells in pancreatic, esophageal and gastric cancer are an independent prognostic factor and are associated with significant elevation of the Th2 cytokine interleukin-13. *Cancer Immunology, Immunotherapy*.

[178] Topalian S., Drake C., Pardoll D. (2015). Immune checkpoint blockade: a common denominator approach to cancer therapy. *Cancer Cell*.

[179] Ott P. A., Hodi F. S., Robert C. (2013). CTLA-4 and PD-1/PD-L1 blockade: New immunotherapeutic modalities with durable clinical benefit in melanoma patients. *Clinical Cancer Research*.

[180] Postow M. A., Callahan M. K., Wolchok J. D. (2015). Immune checkpoint blockade in cancer therapy. *Journal of Clinical Oncology*.

[181] Pardoll D. M. (2012). The blockade of immune checkpoints in cancer immunotherapy. *Nature Reviews Cancer*.

[182] Wang Q., Lin W., Tang X. (2017). The roles of microRNAs in regulating the expression of PD-1/PD-l1 immune checkpoint. *International Journal of Molecular Sciences*.

[183] Mitchell D. A. (2016). MicroRNAs provide a novel pathway toward combinatorial immune checkpoint blockade. *Neuro-Oncology*.

[184] Dudda J. C., Salaun B., Ji Y. (2013). MicroRNA-155 is required for effector cd8+ t cell responses to virus infection and cancer. *Immunity*.

[185] Trotta R., Chen L., Ciarlariello D. (2012). miR-155 regulates IFN-*γ* production in natural killer cells. *Blood*.

[186] Huang C., Li H., Wu W., Jiang T., Qiu Z. (2013). Regulation of miR-155 affects pancreatic cancer cell invasiveness and migration by modulating the STAT3 signaling pathway through SOCS1. *Oncology Reports*.

[187] Fu X., Wen H., Jing L. (2017). MicroRNA-155-5p promotes hepatocellular carcinoma progression by suppressing PTEN through the PI3K/Akt pathway. *Cancer Science*.

[188] Shibuya H., Iinuma H., Shimada R., Horiuchi A., Watanabe T. (2011). Clinicopathological and prognostic value of microRNA-21 and microRNA-155 in colorectal cancer. *Oncology*.

[189] Zhang X. L., Chen J. H., Qin C. K. (2015). MicroRNA-155 expression as a prognostic factor in patients with gallbladder carcinoma after surgical resection. *International Journal of Clinical and Experimental Medicine*.

[190] Tili E., Michaille J.-J., Croce C. M. (2013). MicroRNAs play a central role in molecular dysfunctions linking inflammation with cancer. *Immunological Reviews*.

[191] Rupaimoole R., Wu S. Y., Pradeep S. (2014). Hypoxia-mediated downregulation of miRNA biogenesis promotes tumour progression. *Nature Communications*.

[192] Li Z., Rana T. M. (2014). Therapeutic targeting of microRNAs: current status and future challenges. *Nature Reviews Drug Discovery*.

[193] Ganju A., Khan S., Hafeez B. B. (2017). miRNA nanotherapeutics for cancer. *Drug Discovery Therapy*.

